# Incremental cost-effectiveness of dobutamine stress cardiac magnetic resonance imaging in patients at intermediate risk for coronary artery disease

**DOI:** 10.1007/s00392-014-0793-0

**Published:** 2014-11-14

**Authors:** George Petrov, Sebastian Kelle, Eckart Fleck, Ernst Wellnhofer

**Affiliations:** Department of Internal Medicine/Cardiology, German Heart Institute Berlin, Augustenburger Platz 1, 13353 Berlin, Germany

**Keywords:** Cost-effectiveness analysis, Stress, Magnetic resonance imaging, Coronary angiography, Coronary artery disease

## Abstract

**Aims:**

The effectiveness of stress cardiac magnetic resonance (CMR) as a gatekeeper for coronary angiography (CA) has been established. Level five HTA studies according to the hierarchical model of diagnostic test evaluation are not available.

**Methods:**

This cohort study included 1,158 consecutive patients (mean age 63 ± 11 years, 42 % women) presenting at our institution between January 1, 2003 and December 31, 2004 with suspected coronary artery disease (CAD) for an elective CA. The patients were assessed for eligibility and propensity score matching was applied to address selection bias regarding the patients’ allocation to CMR or direct CA. Median patient follow-up was 7.9 years (95 % CI 7.8–8.0 years). The primary effect was calculated as relative survival difference. The cost unit calculation (per patient) at our institute was the source of costs.

**Results:**

Survival was similar in CMR and CA (*p* = 0.139). Catheterizations ruling out CAD were significantly reduced by the CMR gate-keeper strategy. Patients with prior CMR had significantly lower costs at the initial hospital stay and at follow-up (CMR vs. CA, initial: 2,904€ vs. 3,421€, *p* = 0.018; follow-up: 2,045€ vs. 3,318€, *p* = 0.037). CMR was cost-effective in terms of a contribution of 12,466€ per life year to cover a part of the CMR costs.

**Conclusion:**

Stress CMR prior to CA was saving 12,466€ of hospital costs per life year. Lower costs at follow-up suggest sustained cost-effectiveness of the CMR-guided strategy.

**Electronic supplementary material:**

The online version of this article (doi:10.1007/s00392-014-0793-0) contains supplementary material, which is available to authorized users.

## Introduction

The management of stable patients with suspected coronary artery disease (CAD) is guided by history and evidence of stress-induced myocardial ischemia. The diagnostic accuracy of a stress test varies, depending upon the age, gender and clinical characteristics of the patient, prevalence of CAD in the demographic examined, and modality of the test used. In particular stress imaging is superior to exercise electrocardiogram [1]. The diagnostic accuracy of stress cardiac magnetic resonance (CMR) was found to be higher than stress echocardiography and single photon emission tomography [2–4]. Dobutamine stress CMR (DCMR) is an accurate and safe non-invasive test with high negative predictive value [4, 5]. Several recent long-term follow-up studies demonstrate the safety of a deferral of catheterization in case of negative DCMR [6–8]. Direct catheterization (CA) is still a competitive approach, however, at least in patients with intermediate cardiovascular risk and ambiguous stress electrocardiograms.

CA is still incentivized by the current reimbursement policy in Germany and many other countries. Since long-term outcome and cost data from randomized controlled prospective trials are rarely available when new health technologies emerge, evidence-based reimbursement policy requires retrospective data mining and lags behind medical and technical evolution [9]. High-quality observational data models, simulations and other techniques are commonly used in health technology assessment (HTA) [10, 11]. Studies vary widely regarding imaging modality, methodological approach, control groups and outcome measures and generally adopt the stakeholder perspective of the payer [12, 13]. Moreover, no level five HTA studies according to the hierarchical model of diagnostic test evaluation [14] have been published hitherto.

This paper presents level five HTA data on DCMR based on a long-term follow-up of patients with suspected stable CAD (sCAD) who underwent DCMR and controls with direct CA. We expected that a DCMR-guided approach would be at least as effective as direct CA with respect to survival and more patient-friendly in terms of fewer hospitalizations during follow-up by avoiding direct CA, which is known to have a low diagnostic yield in a routine setting [15].

## Methods

**Fig. 1:**
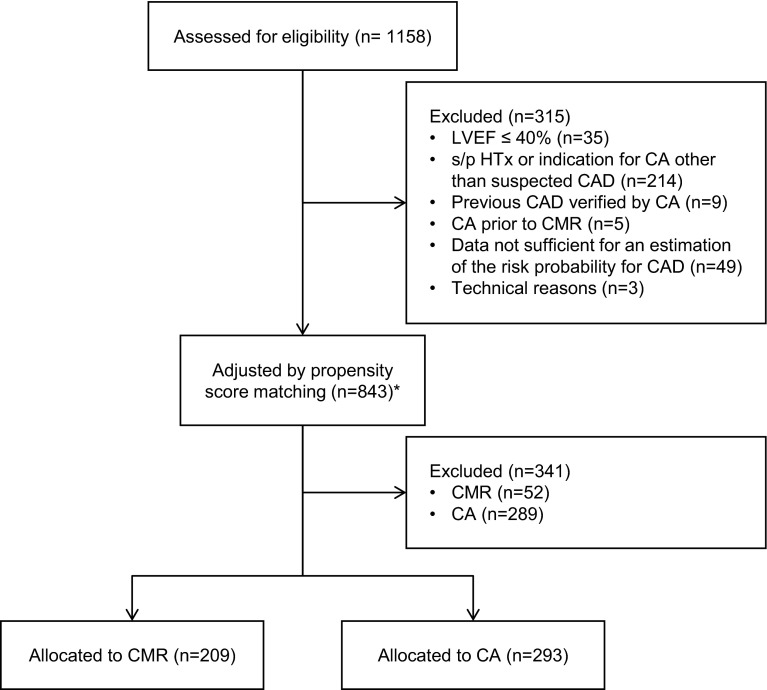
Patient selection. 1,158 consecutive patients referred with suspected sCAD were assessed for eligibility. 843 patients of them remained after exclusion of factors, known to affect the CMR/CA allocation, and were adjusted on their risk probability for CAD by propensity score matching. After matching 502 patients at comparable risk were enrolled. *Asterisk* matching variables: age, gender, LVEF, angina pectoris, hypertension, hyperlipidemia, diabetes mellitus, smoking, ACE inhibitors, β blockers, calcium channel inhibitors, statins. *LVEF* left ventricular ejection fraction, *ACE* angiotensin converting enzyme

This retrospective cohort study is a controlled comparison of two different pathways for managing patients with sCAD and intermediate event risk. The term “intermediate event risk” refers to the risk of annual all-cause mortality of ≥1 but ≤3 % as suggested by the guidelines on the management of sCAD [16]. The source population includes 1,158 consecutive patients referred to the German Heart Institute Berlin between January 1, 2003 and December 31, 2004. Inclusion criteria were sCAD and sufficient data on age, gender, symptoms, cardiovascular (CV) risk factors and medical therapy. Exclusion criteria were known CAD verified by previous angiography, LV ejection fraction (LVEF) ≤40 %, history of cardiac transplantation or an indication different from sCAD for CA. Finally, 843 eligible patients were adjusted for selection bias by propensity score matching and 502 patients remained (CMR: 209 pts. vs. CA: 293 pts.; Fig. 1). The study was approved by the Charité University Hospital Ethics Committee and complies with the principles outlined in the Declaration of Helsinki.

The clinical data were collected from institutional quality assurance and research databases. Missing data were gathered by hospital chart review. The costs data were calculated per patient and hospital stay according to the requirements of the German federal InEK/G-DRG database. Since the InEK calculation was first introduced in 2004 and subsequently modified in 2008, cost data were available in the years 2004–2008 only. The cost calculation method was cost unit accounting based on real processes and expenditures per hospitalized patient. In terms of clean methodology we chose cost contribution accounting as a method to compare both approaches. Discounting or inflation correction was not performed.

Median patient follow-up was 7.9 years (95 % CI 7.8–8.0 years). Primary clinical endpoints were death and the occurrence of cardiac re-hospitalizations. Revascularization by percutaneous coronary intervention (PCI) or coronary artery bypass grafting (CABG) was not treated as endpoint but as covariate to control for its potential impact on survival and costs. Before the study database was closed in December 2011, a query in the digital medical archive was performed to assign unknown deaths and the number of cardiac re-hospitalizations during the entire observation period.

DCMR was performed according to the recommendations of the Society for Cardiovascular Magnetic Resonance [17] using a balanced, fast-field echo sequence with parallel imaging. The imaging methodology has been described in detail previously [6, 18]. Myocardial ischemia was defined as an induced wall motion abnormality or a biphasic response in ≥1 segments of the left ventricle during infusion of dobutamine. Images were analyzed during and immediately after the examination by two experienced investigators without post-processing [6].

The statistical analysis was performed using IBM SPSS Statistics, version 21, and R, version 2.14.2. Clinical data in the tables are presented as mean ± SD or percentages and the cost data as median costs (95 % CI) unless otherwise indicated. Effect estimates were calculated by subtracting the individual event-free survival from the median event-free survival and dividing the difference by the median event-free survival and cost estimates by subtracting the individual costs from the median costs and dividing the difference by the median costs. Incremental cost-effectiveness ratio (ICER) was calculated as.

$$ {\text{ICER}} = \left[ {\frac{{{\text{median costs }}_{{{\text{CMR}}}} {\text{ - median costs }}_{{{\text{CA}}}} }}{{{\text{median survival }}_{{{\text{CMR}}}} {\text{ - median survival }}_{{{\text{CA}}}} }}} \right] $$

and expressed as median cost savings per life year. Unpaired *t* test or Mann–Whitney *U* test were used to compare groups. Categorical variables were tested using Pearson’s *χ* ^2^ test. Cost differences between CMR and CA during follow-up were assessed by two-way ANOVA. Survival was analyzed using Kaplan–Meier and Cox models. A value of *p* < 0.05 was considered statistically significant.

Propensity scores were computed by binary logistic regression with diagnostic path assignment as an outcome variable and age, gender, angina pectoris, CV risk factors and cardiac medications as covariates. A 1:2 nearest neighbor matching algorithm with a caliper of 0.2 of the standard deviation of the logit of the propensity score was chosen to achieve highest possible representativeness and precision. As 20 % of the CMR and 50 % of the CA patients did not meet the matching criteria, they were discarded from the final analysis (Supplement, Figure S1) yielding a final study population of 502 patients. Residual imbalances of covariates after matching were assessed by univariate tests. The largest remaining standardized difference (Cohen’s *d*) was treatment with statins (*d* = −0.09; see supplement, Figure S2). The overall *χ* ^2^ balance test was not significant (*χ* ^2^ = 4.7, *p* = 0.968) and the relative multivariate imbalance L1 measure remained unchanged in the matched sample (0.99 before and after matching), both indicating that matching was successful and improved the overall balance.

## Results

Two hundred and nine from 502 patients with sCAD underwent initial CMR imaging (CMR group). In 14 CMR patients with negative test results (10 % of all negatives) and in 45 CMR patients with positive test results (74 % of all positives) CA was performed. The control group (CA group) comprised 293 patients. Diagnosis of sCAD was functional (exercise-induced wall motion abnormality) in the CMR group and morphological (angiographic stenosis) in the CA group. These different diagnostic modalities resulted in a lower prevalence of CAD in the CMR group (CMR: 29 % vs. CA: 44 %, *p* < 0.001).

The anthropomorphic and clinical characteristics of the CMR and CA groups did not differ significantly after propensity score matching. The patients’ ages and ejection fractions were similar in both groups as was their medical therapies. The Framingham and PROCAM risk scores were also similar as were the prevalence of diabetes mellitus, hypertension, hyperlipidemia and smoking (Table 1).

**Table 1:** Baseline characteristics of the study population

	CMR (*N* = 209)	CA (*N* = 293)	*P*
Age (years)	60 ± 9.6	62 ± 10.5	0.200
Gender (%)			
Male	57	59	0.637
Female	43	41	
LV ejection fraction (%)	59 ± 5.5	59 ± 5.0	0.453
Angina pectoris (%)			
CCS I	27	24	0.715
CCS II	27	25	
CCS III	6	6	
CCS IV	1	0	
Diabetes mellitus (%)	14	17	0.333
Hypertension (%)	72	78	0.122
Hyperlipidemia (%)	57	62	0.255
Smoking (%)	35	30	0.215
Framingham score	8 ± 2.9	9 ± 3.1	0.214
PROCAM score	37 ± 11.6	38 ± 12.1	0.261
ACE inhibitors (%)	57	57	0.990
β-blockers (%)	44	51	0.139
Calcium channel blockers (%)	16	18	0.530
Statins (%)	35	45	0.023

*CMR* cardiac magnetic resonance, *CA* coronary angiography, *CCS* Canadian Cardiovascular Society, *PROCAM* Prospective Cardiovascular Münster Study, *ACE* angiotensin converting enzyme

**Fig. 2:**
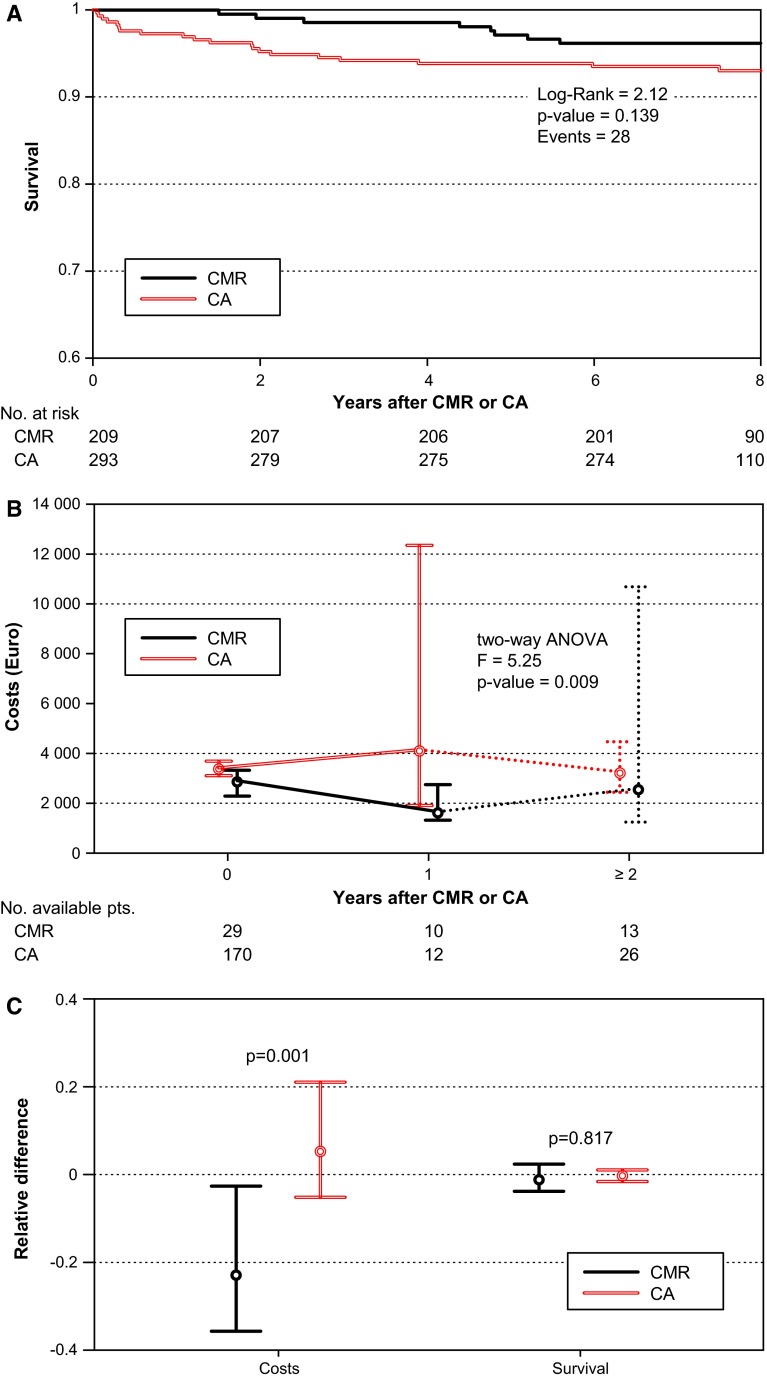
**a** Long-term survival of CMR and CA patients. Survival probability (depicted on the *x-axis*) was cut at 0.6 to visually improve curve’s resolution. The survival difference between CMR and CA was not significant. **b** Temporal dependence of diagnostic path assignment on cost progression. The cost medians with their corresponding 95 % confidence intervals are provided for CMR and CA. During late follow-up (≥2 years) due to sparse data pooled cost estimates (derived from pooled cost data of the years 2006–2008) had to be calculated and are depicted as dashed line. **c** Cost-effectiveness of CMR compared with CA. Median relative differences (see “Methods” for details) with their corresponding 95 % confidence intervals are provided. Pairwise comparison of CMR with CA revealed significant lower overall costs in CMR at similar clinical effectiveness

CMR and CA groups differed in re-hospitalization pattern—in CMR patients predominant ambulatory follow-up was reflected by a larger number of visits to the outpatient department (CMR: 83 % vs. CA: 34 %, *p* = 0.001), whereas CA patients were more often hospitalized (CMR: 35 % vs. CA: 98 %, *p* = 0.001). Death occurred infrequently in both groups (CMR: 4 % vs. CA: 7 %, *p* = 0.149; Table 2). Similar survival was observed in the CMR and CA groups particularly within the first 4 years after study inclusion (*p* = 0.139; Fig. 2a), even after adjustment for revascularization by PCI (HR 1.49, 95 % CI 0.44–5.07, *p* = 0.524) or CABG (HR 0.52, 95 % CI 0.19–1.44, *p* = 0.209) (Supplement, Table S1).

**Table 2:** Clinical endpoints

	CMR (*N* = 209)	CA (*N* = 293)	*P*
CAD (%)	29^a^	44	0.001
PCI (%)	1	21	<0.001
CABG (%)	1	15	<0.001
Death (%)	4	7	0.149
Ambulatory profile			
1–5 outpatient visits (%)	83	34	<0.001
5–10 outpatient visits (%)	12	5	
>10 outpatient visits (%)	3	3	
Hospital profile			
1–5 hospital stays (%)	35	98	<0.001
>5 hospital stays (%)	1	2	

*CMR* cardiac magnetic resonance, *CA* coronary angiography, *CAD* coronary artery disease, *PCI* percutaneous coronary intervention, *CAGB* coronary artery bypass grafting

^a^Diagnosis of “CAD” is either functional (exercise-induced wall motion abnormality) in the CMR group or morphological (angiographic stenosis) in the CA group

The CMR guided approach led to a 72 % reduction in CA utilization, shortened hospital stay length (CMR: 1.22 days, 95 % CI 1.19–1.73 vs. CA: 1.74 days, 95 % CI-1.26–2.08, *p* = 0.022) and produced lower total costs compared to direct CA (CMR: median 2,626€, 95 %-CI 2,193–3,360 vs. CA: median 3,606€, 95 % CI 3,234–4,126 *p* = 0.001). The observed cost reduction occurred at first hospital admission and was maintained at follow-up (Fig. 2b). As suggested by micro-costing data analysis, the increased total costs in the CA group were mainly driven by costs in the cardiology ward and the catheterization laboratory. Differences in costs related to surgery (operating theater, anesthesia, ICU) did not achieve significance possibly due to the small number of CABG procedures. Staff costs and costs allocated for materials and infrastructure were significantly higher in the CA group (Table 3).

**Table 3:** Costs endpoints

	CMR (*N* = 48)	CA (*N* = 181)	*P*
Location of costs			
Cardiology ward (€)	1,337 (1,024–1,420)	1,432 (1,389–1,772)	0.002
Catheterization laboratory (€)	1,016 (864–1,535)	1,308 (1,208–1,542)	0.021
Operating room (€)	4,918 (3,691–6,145)	6,633 (5,420–8,788)	0.257
Anesthesia/ICU (€)	1,250 (1,142–2,306)	2,192 (1,645–2,692)	0.145
Laboratory medicine (€)	105 (61–121)	124 (119–142)	0.001
Radiology (€)	376 (149–710)	351 (238–487)	0.921
Other (€)	301 (24–434)	172 (126–265)	0.631
Type of costs			
Staff (€)	353 (305–397)	462 (423–509)	<0.001
Materials (€)	200 (165–212)	252 (225–261)	<0.001
Infrastructure (€)	492 (436–583)	674 (622–719)	<0.001

*CMR* cardiac magnetic resonance, *CA* coronary angiography, *ICU* intensive care unit, *PCI* percutaneous coronary intervention, *CABG* coronary artery bypass grafting

Comparative cost-effectiveness analysis between CMR and CA showed that the use of CMR was associated with a significant reduction in healthcare costs at similar clinical effectiveness (Fig. 2c). Further ICER indicated that there were 12,466€ cost savings per life year in favor of the CMR-based approach.

## Discussion

In summary, DCMR-guided catheterization in patients at intermediate risk for CAD was at least as effective as direct catheterization in terms of survival and more cost-effective in terms of a substantial contribution margin to cover a part of the CMR costs. As myocardial infarction was not considered as an endpoint the prognostic value regarding ischemic events needs further corroboration. DCMR-guided catheterization was shown to be effective in terms of event-free survival during intermediate [19–21] and long-term [6–8] follow-up in previous studies. Nevertheless, repeated CMR may be recommended after 3 years based on increasing event rates [7]. Comparative effectiveness is sustained during a median follow-up of more than 6 years.

Diagnostic performance of CMR in terms of reclassification of probability of CAD is not an issue in this study [14]. Anyhow, a recent editorial [22] comments on the pitfalls of substituting true diagnosis of functionally significant CAD by gold-standards. The low prevalence of CAD in the cohort reduces the sensitivity of both diagnostic tests (CMR and angiography) and compares well with findings in routine patients [15]. The discrepancy found between functional and morphologic diagnosis of CAD is partially explained by the lack of functional impact of many borderline coronary lesions. An estimate of the rate of functional significant stenosis is given by the rate of interventions multiplied by a factor of 0.63 which is the fraction of functionally significant stenosis in the FAME trial [23]. Finally, the surprisingly low rate of angiographic CAD in patients with positive CMR is not only due to a low prevalence of CAD but also to a very conservative trade-off between sensitivity and specificity in diagnosis (see supplement).

The main cost driver in the CA group was a high rate of catheterizations ruling out significant stenosis. Hospital stays were longer in the CA group and thus costs per patient stay, incurred at the cardiology ward, were increased. The increased costs located at the catheterization laboratory are probably related to an increased rate of PCIs in the CA group. The use of stents in different arms of the FAME trial [23] supports the hypothesis that the lack of information on the functional impact of a lesion may increase the propensity of interventional cardiologists to perform an unnecessary and potentially harmful PCI. Thus, the proof of functional relevance of stenosis is an essential requirement for an indication for revascularization. From the patient’s perspective, this means that invasive interventions and the associated risk of complications might be significantly reduced using an image guided approach. A reduced rate of hospital stays and lower costs at follow-up suggest sustained cost-effectiveness and a patient-friendly ambulatory management profile of the DCMR-guided strategy in agreement with the findings in suspected acute coronary syndrome [24].

As DCMR is not reimbursed in Germany there was no cost calculation available. Moreover, the costs generated in ambulatory patients with negative test in the DCMR pathway depend on the prevalence of the disease. Thus, we decided to calculate the contribution of costs that would be available to cover partial costs of CMR for methodological reasons. In the literature, costs of CMR are generally estimated from reimbursements by the payer on a per patient basis [12, 25–27]. Downstream and secondary costs may be assumed to be lower in patients with a more ambulatory profile in spite of additional imaging costs. Our data suggest that in-hospital cost savings per patient provide a substantial contribution margin to cover imaging costs with no overall cost increase.

Recently there have been several HTA studies on DCMR-gated catheterization [12, 24–29]. However, level five HTA studies demonstrating incremental cost-effectiveness in terms of long-term outcome are not yet available. The published studies vary widely with respect to imaging modality, methodological approach, control groups and outcome measures and generally adopt the stakeholder perspective of the payer. A realistic system for evaluation of emerging technologies is challenged by conflicting needs and expectations of the variety of stakeholders, outdated and distorting incentives set by service valuation and payment, and the lack of a standardized and validated concept of value [30]. Thus, comprehensive HTA analysis should account for stakeholder interests and cost impact [31]. Current reimbursement policies have been shown to be associated with discordant HTA decisions in drug therapy [32]. In particular, the German reimbursement system rewards direct catheterization and discourages an appropriate use of CMR and other imaging technologies as recommended by recent guidelines [16]. Local expertise is supposed to be critical for the choice of imaging modality according to expert consensus and the outcomes presented here imply of course experience in CMR imaging and evaluation.

The International Society for Pharmacoeconomics and Outcomes Research recognizes the necessity and challenge of using secondary data sources, particularly retrospective data, in HTA and specifies principles for good research practice in this field [10]. Every new technology goes through a phase of establishment early in the life cycle. This phase is generally characterized by parallel use of the new technology and standard operating procedures and provides controlled data from the same source population. Secondary data mining and outcome research in these source populations based on a pre-specified hypothesis and statistical matching techniques addressing randomization are valuable sources of evidence beyond randomized controlled trials that are costly and sometimes hampered by discrepancy between real world and highly selected study populations. An increasing number of digital records, big data analyses and advanced statistical approaches [33] facilitate this endeavor. Our study is a single-center retrospective cohort trial based on a pre-specified hypothesis. The data are unique with respect to the duration of follow-up and availability of cost calculation. Moreover, the German Heart Institute is a high volume supra-regional center and was engaged in the early validation of DCMR effectiveness [18]. Outcome differences are comparable to a multicenter analysis [7, 8]. Controlled study design, careful matching, and costs directly calculated from process times and low-level expenses assure the transferability of a contribution margin of in-hospital cost saving by DCMR. Socioeconomic disparities, different reimbursement strategies, jurisdictions and trends in medical treatment strategies may be associated with larger differences in expenses for hospital stays. But, reducing hospitalizations and invasive procedures is expected to cut costs. In agreement with our findings in sCAD, Miller et al. [24] recently demonstrated the potential of DCMR to reduce hospitalizations, invasive procedures, and recurrent tests for ischemia in patients with suspected acute coronary syndrome. The conclusion of this study holds for CMR perfusion imaging, since expenses are similar and effectiveness is comparable [8]. Single photon emission tomography (SPECT) as an imaging modality demonstrated superior cost-effectiveness in the CeCAT trial 2007 [29]. Recent studies, however, found CMR to be more cost-effective than SPECT [25, 27].

## Limitations

We did not include myocardial infarction as an event because extensive manual review of archived electrocardiograms, laboratory data and clinical records would have been necessary to comply with the uniform definition. We fully recognize this limitation. A further limitation is that we do not have data on the cause of death and angina pectoris during follow-up. In the COURAGE study [34], revascularization in patients with functionally significant sCAD had no impact on survival, but reduced angina by a small, but significant, amount that disappeared by 36 months. Thus, the large number of visits to the outpatient department is not fully explained by persistent angina in medically treated sCAD and partially related to a conservative surveillance strategy in these patients. Moreover, inclusion criteria of the COURAGE study do not match with patient selection in this retrospectively sampled cohort. All patients eventually included in the COURAGE study had catheterization. Of course, there are methodological limitations as compared to randomized controlled trials that are inherent in retrospective studies and may not be fully equalized by statistical matching. Moreover, long-term outcome and recentness of management are at odds. Regarding the cost calculation we did not consider discounting or inflation that would have affected the cost differences proportionally. Hospital cost differences were based on cost unit accounting that reflects real processes and resources used per hospitalized patient and should not be seriously affected by prices. Most of the discussed limitations concern the historical data set and not the methodology itself that is likely to profit from growing coverage of digital documentation in heath business and advanced big data mining in the future.

## Electronic supplementary material

Below is the link to the electronic supplementary material.

Supplementary material 1 (DOCX 72 kb)

## Abbreviations

CA: Coronary angiography
CAD: Coronary artery disease
CABG: Coronary artery bypass graft
CI: Confidence interval
CMR: Cardiac magnetic resonance
CV: Cardiovascular
HR: Hazard ratio
HTA: Health technology assessment
ICER: Incremental cost-effectiveness ratio
LV: Left ventricular
PCI: Percutaneous coronary intervention
DRG: Diagnose related groups
InEK: Institut für das Entgeltsystem im Krankenhaus (Agency for calculation of reimbursement of in-hospital treatment)
