# Supplementary material for: Incremental cost-effectiveness of dobutamine stress cardiac magnetic resonance imaging in patients at intermediate risk for coronary artery disease
Source: Clin Res Cardiol. 2014 Nov 14;104(5):401–9. doi: 10.1007/s00392-014-0793-0 (PMC4544498; doi:10.1007/s00392-014-0793-0)
Supplement: Supplementary file 1 — Supplementary material 1 (DOCX 72 kb) [file 392_2014_793_MOESM1_ESM.docx]

**Figure S1.** Dot plot of individual CMR (treatment) or CA (control) patients in either matched or unmatched groups. The propensity scores are plotted on the x-axis. During the matching procedure all patients with dissimilar propensity scores were discarded and an improved balance on the observed pre-test risk probability for CAD was achieved in both groups.

Abbreviations: CMR, cardiac magnetic resonance; CA, coronary angiography; CAD, coronary artery disease;


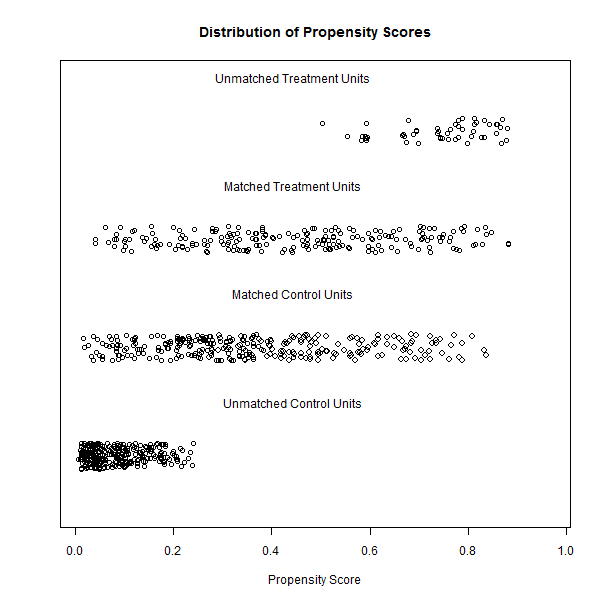


Figure S2. Dot plot of standardized mean differences (Cohen’s d, plotted on the x-axis) for all covariates before and after matching. The largest remaining standardized difference after matching was found at treatment with statins with a value of d = -0.09.

Abbreviations: LVEF, LV ejection fraction; AP, angina pectoris; HTN, hypertension; HLP, hyperlipidemia; DM, diabetes mellitus; ACEI, angiotensin converting enzyme inhibitors; BB, beta blockers; CCI, calcium channel inhibitors;


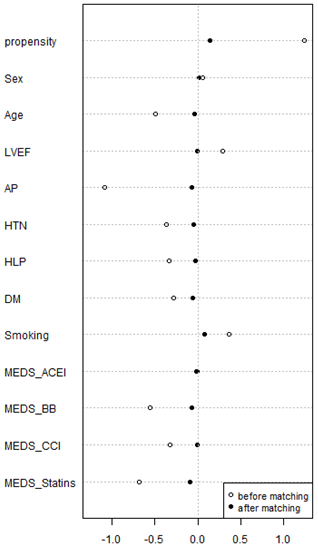


**Table S1-A. Cox regression (crude model)**

|  | HR† | 95% CI | | *p* |
| --- | --- | --- | --- | --- |
|  |  | Lower bound | Upper bound |  |
| Diagnostic path assignment (CMR or CA) | 0.54 | 0.24 | 1.23 | 0.145 |

**Table S1-B. Cox regression (adjusted model)**

|  | HR† | 95% CI | | *p* |
| --- | --- | --- | --- | --- |
|  |  | Lower bound | Upper bound |  |
| Diagnostic path assignment (CMR or CA) | 0.57 | 0.24 | 1.36 | 0.206 |
| PCI | 1.49 | 0.44 | 5.07 | 0.524 |
| CABG | 0.52 | 0.19 | 1.44 | 0.209 |

Abbreviations: HR, hazard ratio; CI, confidence interval; CMR, cardiac magnetic resonance imaging; CA; coronary angiography; PCI, percutaneous coronary intervention; CABG, coronary artery bypass grafting;

**Table S2. Resource allocation**

|  | | CMR (N=48) | CA (N=181) | *p* |
| --- | --- | --- | --- | --- |
| Location of costs | cardiology ward, % | 28% | 25% | 0.686 |
|  | catheterization laboratory, % | 26% | 24% | 0.692 |
|  | operating room/anesthesia/ICU, % | 1% | 4% | **0.045** |
|  | laboratory medicine, % | 26% | 24% | 0.503 |
|  | radiology, % | 10% | 8% | 0.320 |
|  | other, % | 7% | 6% | 0.660 |
| Type of costs | staff, % | 37% | 35% | 0.640 |
|  | materials, % | 39% | 41% | 0.447 |
|  | infrastructure, % | 24% | 24% | 0.726 |

The absolute costs were higher in the CA group, reflecting longer process times. However, no major differences can be found between CMR and CA regarding resource allocation. We provide this additional data for possible transferability demands.

Abbreviations: CMR, cardiac magnetic resonance; CA, coronary angiography; ICU, intensive care unit;

**Table S3. Severity of CAD**

|  | CMR (n=209) | CA (n=293) | *p* |
| --- | --- | --- | --- |
| No evidence of CAD, % | 91 | 56 | < 0.0001 |
| Lumen obstruction ≤50 per cent, % | 0 | 7 | < 0.0001 |
| One- or two- vessels disease, % | 5 | 23 | < 0.0001 |
| Three- vessels disease, % | 2 | 10 | < 0.0001 |
| Left main disease, % | 2 | 4 | 0.018 |

The surprisingly low rate of angiographic CAD in patients with positive CMR is not only due to a low prevalence of CAD but also to a very conservative trade-off between sensitivity and specificity in diagnosis. Even patients with suspected wall motion abnormalities on the CMR exam underwent angiography at that time, when CMR emerged as new imaging modality of cardiac ischemia. This risk management strategy at the cost of sensitivity was associated with a high specificity. None of the patients who underwent catheterization despite a negative CMR was re-vascularized without negative impact on outcome.

Post-hoc analysis of severity of angiographic findings suggests that despite of propensity score matching, the patients in the CA group had more severe disease. There are some methodological shortcomings of this comparison, however. Particularly angiographic diagnosis was not based on QCA but visual and thus probably biased evaluation.

Abbreviations: CMR, cardiac magnetic resonance; CA, coronary angiography; CAD, coronary artery disease;
